# Towards sustainable architecture: Enhancing green building energy consumption prediction with integrated variational autoencoders and self-attentive gated recurrent units from multifaceted datasets

**DOI:** 10.1371/journal.pone.0317514

**Published:** 2025-04-25

**Authors:** Qing Zeng, Fang Peng, Xiaojuan Han

**Affiliations:** College of Architecture and Urban Planning, Hunan City University, Yiyang, China; University of Southern California, UNITED STATES OF AMERICA

## Abstract

Global awareness of sustainable development has heightened interest in green buildings as a key strategy for reducing energy consumption and carbon emissions. Accurate prediction of energy consumption plays a vital role in developing effective energy management and conservation strategies. This study addresses these challenges by proposing an advanced deep learning framework that integrates Time-Dependent Variational Autoencoder (TD-VAE) with Adaptive Gated Self-Attention GRU (AGSA-GRU). The framework incorporates self-attention mechanisms and Multi-Task Learning (MTL) strategies to capture long-term dependencies and complex patterns in energy consumption time series data, while simultaneously optimizing prediction accuracy and anomaly detection. Experiments on two public green building energy consumption datasets validate the effectiveness of our proposed approach. Our method achieves a prediction accuracy of 93.2%, significantly outperforming traditional deep learning methods and existing techniques. ROC curve analysis demonstrates our model’s robustness, achieving an Area Under the Curve (AUC) of 0.91 while maintaining a low false positive rate (FPR) and high true positive rate (TPR). This study presents an efficient solution for green building energy consumption prediction, contributing significantly to energy conservation, emission reduction, and sustainable development in the construction industry.

## 1 Introduction

### 1.1 Background

Climate change presents a global challenge that has elevated the importance of energy conservation and emission reduction in the construction industry. The World Economic Forum reports that building operations and construction activities jointly contribute to approximately 40% of global energy-related CO_2_ emissions. Supporting these findings, the United Nations Environment Programme (UNEP) documented that in 2021, the building and construction sector consumed over 34% of global energy and generated approximately 37% of energy and process-related CO_2_ emissions. Therefore, precise prediction of building energy consumption plays a vital role in energy control, design optimization, retrofit assessment, and comprehensive energy management strategies. These predictive models serve as essential tools for improving energy efficiency, achieving carbon neutrality, and promoting sustainable green building development through optimized energy management systems.

Recent advances in information technology and smart systems have revolutionized data-driven methods for predicting building energy consumption. In a comprehensive review, Bourdeau et al. examined data-driven technologies for building energy modeling and highlighted the potential of machine learning in developing adaptive energy forecasting methods [1]. The combination of intelligent algorithms, especially deep learning approaches, with energy consumption data analysis has proven effective in providing scientific decision support, while optimizing both operational efficiency and economic outcomes.

Based on a comparative analysis of intelligent technologies, Amber et al. found that artificial neural networks outperformed other methods in predicting building electrical consumption [2]. Nevertheless, accurate prediction remains challenging due to complex energy consumption patterns influenced by building characteristics, occupant behavior, and environmental conditions. Amasyali and El-Gohary, along with Ahmad et al., validated data-driven models’ effectiveness while identifying critical research gaps and future research directions [3,4].

This study presents an innovative framework for building energy consumption prediction by integrating Variational Autoencoders (VAE) with Gated Recurrent Units (GRU), enhanced through self-attention mechanisms. The proposed approach addresses both temporal patterns and nonlinear relationships in building energy consumption. The integration of VAE and GRU enables effective capture of temporal data patterns, while self-attention mechanisms enhance feature detection, resulting in robust prediction performance across different building energy systems. The research advances green building development by supporting energy conservation and sustainable development in the construction industry.

### 1.2 Literature review

Green buildings have emerged as a critical solution for environmental sustainability through efficient energy management. According to recent studies, understanding and optimizing building energy consumption has become increasingly vital [7,8]. Qiao and Liu developed quantitative methods to assess greening impacts on building energy consumption, providing empirical support for urban greening policies [5]. While their approach offered valuable insights, it lacked consideration of temporal dependencies in energy consumption patterns. Ali highlighted how green buildings reduce environmental impact through energy-efficient design principles and sustainable practices [6], though the study primarily focused on design aspects without addressing operational optimization. Building upon this foundation, Li et al. implemented the Isolation Forest algorithm for anomaly detection in energy consumption patterns [9], but their single-task approach limited the model’s ability to capture complex relationships between different aspects of building performance. Rameshwar et al. examined both passive and active technologies in green building design [10], yet their analysis did not fully address the integration of these technologies with advanced prediction methods. Notably, Xue and Zhao demonstrated that effective energy consumption monitoring could achieve approximately 8% annual energy savings through optimized circuit design [11], although their approach lacked adaptability for different building types.

The advancement of deep learning has transformed energy consumption prediction methodologies. Almalaq and Zhang integrated genetic algorithms with Long Short-Term Memory networks to enhance prediction accuracy [12], but their method exhibited high computational complexity and limited interpretability. Wang et al. provided a comprehensive review of deep learning applications in renewable energy prediction [13], emphasizing the need for more sophisticated feature extraction mechanisms. Significant advances include Jana et al.’s granular approach combining Maximum Overlap Discrete Wavelet Transform with LSTM networks [14], although their model showed limitations in long-term dependency capture. Gao et al. developed sequence-to-sequence models and attention-enhanced CNNs [15], but their approach lacked multi-task learning capabilities. Liu et al. explored Deep Reinforcement Learning applications [16], yet encountered difficulties in real-time adaptation. Gao and Ruan developed interpretable LSTM-based models with self-attention mechanisms [17], though their method did not fully address the integration of anomaly detection. Recent work by Soudaei et al. introduced automated architecture optimization using Differential Evolution algorithms [18], but their approach required extensive computational resources for architecture search.

Recent research has particularly focused on incorporating sophisticated mechanisms to improve prediction accuracy. Ju et al. demonstrated the effectiveness of combining self-attention mechanisms with multi-task learning for short-term power output prediction [19], though their model showed limitations in handling diverse building types. Bu and Cho developed a multi-head attention model integrated with convolutional recurrent neural networks [20], but their approach lacked efficient feature selection mechanisms. Gao and Ruan enhanced model interpretability through attention-based feature analysis [17], yet their method didn’t fully address the temporal aspects of energy consumption. Guo et al. introduced an innovative external attention mechanism using learnable shared memories [21], though their approach required significant memory resources. Building upon these advances, Qiao et al. developed a multi-level attention mechanism for capturing complex feature correlations [22], but their model showed limitations in handling anomaly detection tasks.

Despite these advances, several fundamental challenges persist in green building energy consumption prediction. Although deep learning methods generally outperform traditional approaches, they encounter challenges in three key aspects. First, effectively capturing both high-dimensional feature representations and complex temporal dependencies poses a substantial challenge, as existing methods often struggle to balance feature extraction capabilities with temporal modeling. Second, while various deep learning architectures have been proposed for temporal modeling, they frequently exhibit limitations in capturing long-term dependencies and dynamically focusing on key temporal patterns, affecting prediction accuracy especially in complex building scenarios. Third, current single-task approaches fail to leverage the complementary information between different prediction tasks, potentially overlooking valuable insights that could enhance overall performance. These limitations motivate the proposed approach, which addresses these challenges through an innovative integration of TD-VAE for robust temporal dependency modeling, AGSA-GRU for adaptive temporal pattern capture, and a multi-task learning framework that optimizes the synergy between prediction and anomaly detection tasks.

**Table 1:** Analysis of current challenges and research opportunities in building energy consumption prediction.

Research aspect	Current challenges	Research opportunities
Temporal feature learning	Traditional LSTM models [12,14] face challenges in simultaneously handling high-dimensional input features and complex temporal dependencies in building energy data	Exploration of hybrid architectures combining variational approaches with temporal modeling to better capture both feature representation and time dependencies
Long-term dependency modeling	Current RNN-based approaches [26,27] show limitations in capturing long-range temporal dependencies and dynamically focusing on key temporal patterns	Development of enhanced attention mechanisms that can adaptively adjust temporal focus while maintaining computational efficiency
Task integration	Existing single-task methods [19,20] fail to leverage the complementary information between prediction and anomaly detection tasks, leading to suboptimal performance	Investigation of multi-task frameworks that can effectively balance and optimize the synergy between different tasks while sharing learned representations

Based on the comprehensive literature analysis summarized in Table 1, this study identified three critical gaps in green building energy consumption prediction: optimizing model performance while maintaining computational efficiency, effectively integrating multi-task learning capabilities, and improving model interpretability. The proposed framework addresses these challenges through an innovative integration of VAE-GRU architecture with self-attention mechanisms, providing enhanced feature learning capabilities while offering interpretable insights into the prediction process. This approach not only advances the current state of research but also provides practical solutions for real-world applications in green building energy management.

### 1.3 Our contributions

- This study integrates the Temporal Dependency-enhanced Variational Autoencoder (TD-VAE) and the Adaptive Gated Self-Attention GRU (AGSA-GRU), effectively addressing the challenges of long-term temporal dependencies and complex feature processing in green building energy consumption prediction.
- By introducing a Multi-Task Learning (MTL) strategy, which co-optimizes energy consumption prediction and anomaly detection tasks, this research enhances both the model’s understanding of data structures and its predictive performance and robustness.
- The integration of TD-VAE, AGSA-GRU, and multi-task learning strategy proposed in this study significantly improves the accuracy and efficiency of building energy consumption prediction while providing effective support for energy optimization management.

## 2 Our method

### 2.1 Problem description

Considering a set of *n* buildings in enhancing the accuracy and efficiency of green building energy consumption prediction using deep learning technology, each building *i* has its specific energy consumption dataset $D_{i}$. The energy consumption prediction problem for building *i* is defined as predicting future energy consumption based on its historical energy consumption data $D_{i}$. Specifically, for each time point *t*, let $x_{t}$ be the input feature vector (such as temperature, humidity, time, etc.), and $y_{t}$ the actual energy consumption value. The goal is to find model parameters $w\in {\mathbb{R}}^{m}$ so that the model can accurately predict energy consumption $y_{t}$ based on input $x_{t}$.

This problem can be formalized as the following optimization problem:


min ⁡ w∈ℝmF(w)=1n∑i=1nfi(w) (1)

where, $f_{i}(w)$ is the loss function for building *i*, defined as:


$$\begin{array}{ccc} f_{i}(w)=\frac{1}{\left|D_{i}\right|}\underset{(x_{t},y_{t})\in D_{i}}{\sum }L(y_{t},{ŷ}_{t}(w;x_{t})) & & \end{array}$$ (2)

**Fig 1:**
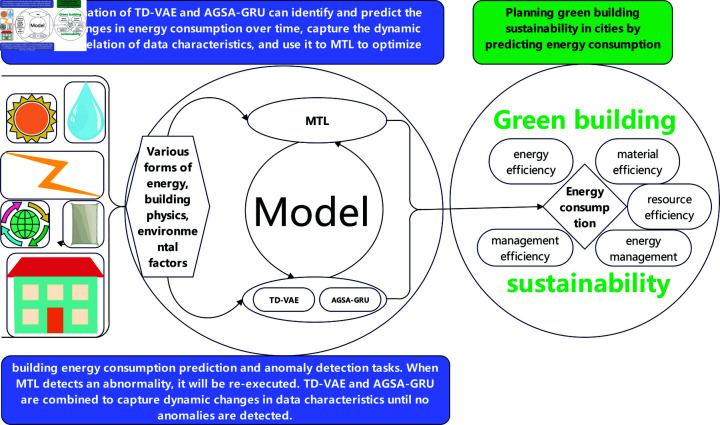
The flow chart of the entire experiment.

$L(y_{t},{ŷ}_{t}(w;x_{t}))$ is the loss function, quantifying the error between model prediction ${ŷ}_{t}(w;x_{t})$ and actual energy consumption $y_{t}$. $\left|D_{i}\right|$ is the number of samples in the dataset $D_{i}$ of building *i*. The challenges include:


max ⁡ w∈ℝm ∑i=1nVar(fi(w)) (3)

where, $\text{Var}(f_{i}(w))$ represents the variance of model predictions across different building datasets, reflecting the model’s generalization ability and stability across various building environments. We will further explore how to effectively process time series data through deep learning models, and leverage specific energy consumption patterns of buildings to enhance prediction accuracy and efficiency. The core challenge lies in designing models that can capture the dynamic characteristics of building energy consumption and handling the nonlinearity and high-dimensionality of building energy consumption data. To address this issue, we introduce our model process as shown in Fig 1.

### 2.2 Motivation for temporal dependency-enhanced variational autoencoder (TD-VAE)

- Traditional building energy consumption prediction methods, including statistical approaches and basic machine learning models, exhibit limitations in capturing intrinsic data characteristics. Specifically, these methods struggle with modeling long-term time dependencies and processing high-dimensional, nonlinear features in building energy data [23–25]. Such limitations directly affect prediction reliability, particularly in complex green building systems where energy patterns vary significantly over time.
- To address these challenges effectively, we propose the Temporal Dependency-enhanced Variational Autoencoder (TD-VAE), which strategically combines VAE and GRU architectures. The VAE component enables robust feature extraction and dimensionality reduction, while the GRU structure captures temporal dependencies. This integration is particularly effective for green building scenarios as the VAE efficiently handles the high-dimensional building sensor data through its encoding mechanism, while the GRU captures both short-term and long-term energy consumption patterns. The architecture adapts to varying operational conditions through its flexible structure, creating synergy between components that enables superior capture of complex energy consumption patterns and enhanced prediction accuracy.

### 2.3 Mathematical derivation of temporal dependency-enhanced variationalautoencoder (TD-VAE)

The temporal dependency-enhanced variational lower bound (TD-ELBO) for the objective function $f_{i}(w)$, to achieve time series modeling of energy consumption data for each building *i*:


LTD(𝜃,ϕ;{xti},{yti})= ∑t=1T (𝔼qϕ(zti|xti)[log ⁡ p𝜃(yti|zti,xti)]−βDKL(qϕ(zti|xti)||p(zti|zt−1i))) (4)

where $q_{\phi }(z_{t}^{i}|x_{t}^{i})$ is the posterior distribution of latent variable $z_{t}^{i}$ given input $x_{t}^{i}$, $p_{𝜃}(y_{t}^{i}|z_{t}^{i},x_{t}^{i})$ is the probability distribution of output $y_{t}^{i}$ given latent variable $z_{t}^{i}$ and input $x_{t}^{i}$, $D_{KL}$ is the Kullback-Leibler divergence, *β* is a hyperparameter balancing the reconstruction error and KL divergence.

To model the time-dependency of latent variable $z_{t}^{i}$, we use a Gated Recurrent Unit (GRU) to update $z_{t}^{i}$:


$$\begin{array}{ccc} z_{t}^{i}=GRU(z_{t-1}^{i},x_{t}^{i};\phi ) & & \end{array}$$ (5)

where *GRU* denotes the GRU model, and *ϕ* is the model parameters. This enables the model to capture and utilize the time-dependency of energy consumption data.

We define the KL divergence term of TD-VAE to consider time-dependency, using the conditional prior $p(z_{t}^{i}|z_{t-1}^{i})$ to describe the temporal evolution of latent states:


DKL(qϕ(zti|xti)||p(zti|zt−1i))= ∑t=1T𝔼qϕ(zti|xti) [log ⁡ qϕ(zti|xti)p(zti|zt−1i)] (6)

where $p(z_{t}^{i}|z_{t-1}^{i})$ is the conditional prior distribution of $z_{t}^{i}$, indicating the distribution of $z_{t}^{i}$ given $z_{t-1}^{i}$.

To optimize TD-VAE, we propose an optimization strategy based on Stochastic Gradient Descent (SGD), specifically, optimizing the objective function $L_{TD}$ with respect to parameters *𝜃* and *ϕ*. Through an alternating optimization strategy, first fixing *𝜃* to optimize *ϕ*, then fixing *ϕ* to optimize *𝜃*, until convergence:


𝜃(k+1),ϕ(k+1)= arg ⁡ max ⁡ 𝜃,ϕLTD(𝜃(k),ϕ(k);{xti},{yti}) (7)

where *k* is the iteration count.

To further enhance the model’s generalization ability and stability, we consider incorporating regularization terms $\lambda ∥𝜃{∥}_{2}^{2}+\gamma ∥\phi {∥}_{2}^{2}$ into the optimization objective, where *λ* and *γ* are regularization coefficients.

**Problem 1.** How to quantify and maximize the capture of time-dependency within the TD-VAE model and assess its impact on the model’s predictive performance?

The TD-VAE model predicts future energy consumption based on historical data. The model explicitly models time-dependency through a KL divergence term, where the parameter *β* controls the strength of dependency.

Definition: Time-dependency capture capability $C_{\text{TD}}(\beta )$ quantifies the model’s understanding of time series dependencies. Predictive performance is measured through Mean Squared Error (MSE), evaluating the model’s prediction accuracy.

The goal is to find the optimal $\beta^{*}$ value that minimizes prediction error MSE while maintaining time-dependency capture capability above a certain threshold *δ*. We introduce an optimization problem with a constraint to represent this:


β∗= arg ⁡ min ⁡ β {MSE(β):CTD(β)≥δ} (8)

$\text{MSE}(\beta )=\frac{1}{T}\sum_{t=1}^{T}{\left(y_{t}-{ŷ}_{t}\right)}^{2}$ is the objective function we aim to minimize, while $C_{\text{TD}}(\beta )=\sum_{t=2}^{T}{𝔼}_{q_{\phi }(z_{t}|x_{t})}\left[D_{KL}(q_{\phi }(z_{t}|x_{t})||p(z_{t}|z_{t-1}))\right]$ represents the time-dependency capture capability, which must satisfy the constraint  ≥ *δ*. This expression directly relates the balance between optimizing model predictive performance and capturing time-dependency.

**Theorem 1** (Time-dependency Capture and Prediction Error Optimization). In discussing the relationship between time series dependency capture and predictive performance in the TD-VAE model, we define an optimization problem aimed at finding the optimal $\beta^{*}$ value that exceeds a time-dependency capture threshold *δ* and minimizes prediction error:


β∗= arg ⁡ min ⁡ β {1T∑t=1T(yt−ŷt)2}subject to∑t=2T𝔼qϕ(zt|xt) [DKL(qϕ(zt|xt)||p(zt|zt−1))]≥δ (9)

**Corollary 1.** In a deeper analysis of the Temporal Dependency-enhanced Variational Autoencoder (TD-VAE) model, we aim to prove the existence of a delicate balance mechanism within the model. This mechanism optimizes its predictive performance while maintaining the capability to capture time-dependency. Integrating the concept of Temporal Dependency-enhanced Evidence Lower Bound (TD-ELBO), the tuning role of the *β* parameter, quantitative analysis of time-dependency capture capability, and consideration of prediction error, we propose the following composite corollary formula:


β∗= arg ⁡ min ⁡ β∈[βmin ⁡ ,βmax ⁡ ] {1T∑t=1T(yt−ŷt)2+λ (max ⁡  {0,δ−∑t=2T𝔼qϕ(zt|xt) [DKL(qϕ(zt|xt)||p(zt|zt−1))]})2 } (10)

This formula comprehensively demonstrates how, in the TD-VAE model, precise adjustment of the *β* value, while ensuring the time-dependency capture capability exceeds a predefined threshold *δ*, achieves minimization of prediction error MSE. Within this framework, *λ* acts as a regularization parameter, balancing the relationship between time-dependency capture and minimization of prediction error.

### 2.4 Motivation for adaptive gated self-attention GRU (AGSA-GRU)

- Although GRUs and RNNs significantly perform in handling short-term dependencies in time series, they fall short in capturing long-term dependencies, understanding complex dynamic characteristics, and automatically recognizing key time steps. This is primarily due to the inherent limitations of these model structures, especially in tasks involving high-dimensional and nonlinear features, such as building energy consumption prediction [26–28].
- To complement and enhance the temporal modeling capabilities of TD-VAE, we introduce the Adaptive Gated Self-Attention GRU (AGSA-GRU). This model integrates a self-attention mechanism with GRU architecture through an adaptive gating system, enabling dynamic adjustment of temporal focus based on data characteristics. The combination of TD-VAE’s robust temporal dependency modeling and AGSA-GRU’s adaptive attention mechanism creates a comprehensive framework for accurate and efficient prediction.

### 2.5 Mathematical derivation of adaptive gated self-attention GRU (AGSA-GRU)

We define the model framework for Adaptive Gated Self-Attention GRU (AGSA-GRU). Considering the output from the TD-VAE model as input to AGSA-GRU, we introduce the following definition:


$$\begin{array}{ccc} h_{t}^{i}=AGSA\text{-}GRU(h_{t-1}^{i},x_{t}^{i};\Psi ) & & \end{array}$$ (11)

where $h_{t}^{i}$ represents the hidden state at time *t*, *Ψ* denotes the parameter set of AGSA-GRU, and $x_{t}^{i}$ is the input feature vector.

The adaptive gating mechanism of AGSA-GRU can be mathematically described as follows:


$$\begin{array}{ccc} z_{t}^{i} & =\sigma (W_{z}\cdot [h_{t-1}^{i},x_{t}^{i}]+b_{z}) & \end{array}$$ (12)

$$\begin{array}{ccc} r_{t}^{i} & =\sigma (W_{r}\cdot [h_{t-1}^{i},x_{t}^{i}]+b_{r}) & \end{array}$$ (13)

$$\begin{array}{ccc} {\tilde{h}}_{t}^{i} & =\tanh(W\cdot [r_{t}^{i}⊙h_{t-1}^{i},x_{t}^{i}]+b) & \end{array}$$ (14)

$$\begin{array}{ccc} h_{t}^{i} & =(1-z_{t}^{i})⊙h_{t-1}^{i}+z_{t}^{i}⊙{\tilde{h}}_{t}^{i} & \end{array}$$ (15)

where *σ* is the sigmoid activation function,  ⊙  denotes element-wise multiplication, $W_{z},W_{r},W,b_{z},b_{r},b$ are model parameters, and $z_{t}^{i}$ and $r_{t}^{i}$ are the update and reset gates, respectively.

The introduction of the self-attention mechanism further enhances the model’s capability to capture important features in the time series:


$$\begin{array}{ccc} A_{t}^{i} & =\text{softmax}(W_{a2}\cdot \tanh(W_{a1}\cdot H_{t}^{i}+b_{a1})+b_{a2}) & \end{array}$$ (16)

$$\begin{array}{ccc} {ĥ}_{t}^{i} & =A_{t}^{i}⊙H_{t}^{i} & \end{array}$$ (17)

where $H_{t}^{i}$ is a set of hidden states within a time window, $A_{t}^{i}$ is the self-attention weight, ${ĥ}_{t}^{i}$ is the hidden state weighted by self-attention, and $W_{a1},W_{a2},b_{a1},b_{a2}$ are parameters of the self-attention mechanism.

Combining the outputs of AGSA-GRU and TD-VAE, we define a joint optimization objective:


LAGSA(Θ,Ψ;{xti},{yti})= ∑t=1T (log ⁡ pΘ(yti|ĥti,xti)−λDKL(pΘ(ĥti|xti)||q Ψ(ĥti|ht−1i))) (18)

where *λ* is a hyperparameter used to balance the reconstruction error and KL divergence, *Θ* and *Ψ* represent the parameter sets of TD-VAE and AGSA-GRU, respectively.

Optimizing the parameters involved in the AGSA-GRU model involves using the Stochastic Gradient Descent (SGD) method, iteratively updating parameters:


Θ(k+1),Ψ(k+1)= arg ⁡ min ⁡ Θ,ΨLAGSA(Θ(k),Ψ(k);{xti},{yti}) (19)

To improve the stability and generalization ability of the model, regularization terms are further introduced:


Θ(k+1),Ψ(k+1)= arg ⁡ min ⁡ Θ,Ψ (LAGSA(Θ(k),Ψ(k);{xti},{yti})+μ∥Θ∥22+ν∥Ψ∥22) (20)

where *μ* and *ν* are the coefficients for the regularization terms of TD-VAE and AGSA-GRU, respectively. Through the mathematical derivation above, we can see that the AGSA-GRU model not only considers the dynamic characteristics of time series data but also effectively enhances the feature capturing capability through the self-attention mechanism, thereby improving the accuracy and efficiency of green building energy consumption prediction.

**Problem 2.** In the AGSA-GRU model, the self-attention mechanism enhances the model’s predictive capability by assigning different weights to states at different time steps. The key is how to precisely quantify and optimize the contribution of the self-attention mechanism to improve predictive accuracy.

The core problem is how to adjust the AGSA-GRU model parameters *Ψ* to maximize the efficiency of the self-attention mechanism $E_{\text{att}}(\Psi )$ while ensuring the model’s predictive accuracy does not decrease?

We define the optimization problem of the self-attention mechanism efficiency, aiming to maximize the self-attention efficiency while controlling the prediction error:


Ψ∗= arg ⁡ max ⁡  Ψ (1T∑t=1T (∑j=1NAt,ji⋅ log ⁡  (At,jiA¯ji)))subject toMSEval(Ψ)≤𝜖 (21)

By solving this optimization problem, we aim to find a balance point, that is, maximizing the efficiency of the self-attention mechanism without sacrificing predictive accuracy.

**Theorem 2** (Optimization Trade-off Framework for Self-Attention Mechanism). In studying the AGSA-GRU model, we explore how the self-attention mechanism enhances the capability for time series prediction and refine the relationship between the self-attention efficiency $E_{\text{att}}(\Psi )$ and model predictive accuracy. The goal is to find a set of parameters $\Psi^{*}$, by optimizing the trade-off between self-attention efficiency and predictive accuracy, considering time-dependency, to form an optimization problem:


Ψ∗= arg ⁡ min ⁡  Ψ {− (−1T∑t=1T ∑j=1NAt,ji log ⁡ At,ji)+ρ⋅ (1T∑t=1Tωt⋅(yt−ŷt)2)+λ⋅DTD(Ψ)} (22)

where$E_{\text{att}}(\Psi )$ quantifies how the model diversely considers the importance of different time points, ${\text{MSE}}_{\text{val}}(\Psi )$ evaluates the model’s predictive accuracy through weighted mean squared error, and $D_{\text{TD}}(\Psi )$ accounts for the impact of time dependency. *λ* and *ρ* are regularization coefficients, used to balance the relationship between self-attention efficiency, predictive accuracy, and time dependency capturing.

**Corollary 2.** In the study of the AGSA-GRU model, we propose a comprehensive objective function *H* ( *Ψ* ) , to minimize the model parameters *Ψ*, while optimizing the prediction error, enhancing the efficiency of the self-attention mechanism, and deepening the understanding of time-dependency in time series data:


Ψ∗=arg ⁡ min ⁡  Ψ {MSEval(Ψ)−ξ (−λeff∑t=1T ∑j=1Nωt,j⋅(At,ji−A¯ji)2⋅ΔMSE,ti )+α∥Ψ∥22+β∑t=1T ( ∑j=1N|At,ji−A¯ji|p )+γ1T−1∑t=2Tσt⋅DKL(qΨ(zt|xt)||p(zt|zt−1)) } (23)

where *ξ*, *α*, *β*, and *γ* are coefficients adjusting the importance of each term, ${\text{MSE}}_{\text{val}}(\Psi )$ assesses the model’s predictive accuracy, $F_{\text{eff}}(\Psi )$ and *Ω* ( *Ψ* )  represent the self-attention efficiency and model regularization terms, respectively, $G_{\text{TD}}(\Psi )$ measures the effect of time dependency modeling. By minimizing *H* ( *Ψ* ) , we aim to find the optimal model parameters $\Psi^{*}$, achieving the minimization of prediction error, maximization of self-attention mechanism efficiency, and deepened understanding of time dependency, demonstrating the core role of the self-attention mechanism in the AGSA-GRU model and its new perspective on handling time-dependency in time series data.

### 2.6 Motivation for multi-task learning

- Model optimization under single-task learning frameworks often overlooks the correlations and complementary information between different tasks, especially in the field of building energy consumption prediction, limiting model performance. Additionally, single-task approaches may ignore important information from one task while capturing characteristics of another, affecting the model’s generalization ability and practicality [15,29,30].
- To overcome these limitations, we implement a Multi-task Learning (MTL) strategy that simultaneously optimizes building energy consumption prediction and anomaly detection. This approach enables parallel processing of multiple tasks within a unified model framework, facilitating the learning of shared representations across tasks. Specifically, the anomaly detection task helps identify irregular energy consumption patterns and potential system malfunctions, which in turn guides the prediction task to generate more reliable forecasts by giving less weight to anomalous data points during training.

### 2.7 Mathematical derivation for multi-task learning

In the Multi-task Learning (MTL) framework, we aim to minimize a joint loss function that integrates the loss functions of energy consumption prediction tasks and anomaly detection tasks. Let the model parameters be *Θ*, the loss function for energy consumption prediction task as $L_{pred}(y_{t}^{i},{\hat{y}}_{t}^{i};\Theta )$, and the loss function for the anomaly detection task as $L_{anom}(z_{t}^{i},{\hat{z}}_{t}^{i};\Theta )$, we can define the joint loss function *J* as follows:


$$\begin{array}{ccc} J(\Theta )=\alpha L_{pred}(y,\hat{y};\Theta )+(1-\alpha )L_{anom}(z,\hat{z};\Theta ) & & \end{array}$$ (24)

where *α* ∈ [ 0 , 1 ]  is a hyperparameter used to balance the two tasks.

When optimizing *J* ( *Θ* ) , the Stochastic Gradient Descent (SGD) method is used, and the model parameters are updated as follows:


$$\begin{array}{ccc} \Theta_{new}=\Theta_{old}-\eta \nabla J(\Theta ), & & \end{array}$$ (25)

where *η* denotes the learning rate.

First, for $L_{pred}$, considering its Taylor second-order expansion around *Θ*, assuming $L_{pred}$ satisfies *β*-smooth and *μ*-strongly convex conditions, then:


$$\begin{array}{ccc} L_{pred}(\Theta +\Delta \Theta )\approx L_{pred}(\Theta )+\nabla L_{pred}{\left(\Theta \right)}^{T}\Delta \Theta +\frac{1}{2}\Delta \Theta^{T}\nabla^{2}L_{pred}(\Theta )\Delta \Theta & & \end{array}$$ (26)

where *ΔΘ* is the increment of *Θ*.

To understand the gradient structure of *J* ( *Θ* )  better, we expand $L_{pred}$ and $L_{anom}$ using Taylor series and introduce Lagrange multipliers for constrained optimization, obtaining:


$$\begin{array}{ccc} \begin{array}{ccc} \nabla J(\Theta ) & =\alpha \left(\nabla L_{pred}(y,\hat{y};\Theta )+\overset{m}{\underset{i=1}{\sum }}\lambda_{i}\frac{\partial g_{i}(\Theta )}{\partial \Theta }\right) & \\ & \;+(1-\alpha )\left(\nabla L_{anom}(z,\hat{z};\Theta )+\overset{n}{\underset{j=1}{\sum }}\mu_{j}\frac{\partial h_{j}(\Theta )}{\partial \Theta }\right) \end{array} & & \end{array}$$ (27)

$$\begin{array}{ccc} \begin{array}{ccc} \nabla J(\Theta )= & \;\alpha \left(\nabla L_{pred}(y,\hat{y};\Theta )+\overset{m}{\underset{i=1}{\sum }}\lambda_{i}\nabla_{\Theta }g_{i}(\Theta )+\frac{1}{2}\overset{m}{\underset{i=1}{\sum }}\overset{m}{\underset{j=1}{\sum }}\lambda_{i}\lambda_{j}\nabla_{\Theta }^{2}(g_{i}(\Theta )g_{j}(\Theta ))\right) & \\ & +(1-\alpha )\left(\nabla L_{anom}(z,\hat{z};\Theta )+\overset{n}{\underset{k=1}{\sum }}\mu_{k}\nabla_{\Theta }h_{k}(\Theta )+\frac{1}{2}\overset{n}{\underset{k=1}{\sum }}\overset{n}{\underset{l=1}{\sum }}\mu_{k}\mu_{l}\nabla_{\Theta }^{2}(h_{k}(\Theta )h_{l}(\Theta ))\right)\\ & +\frac{1}{2}\alpha (1-\alpha )\left(\overset{m}{\underset{i=1}{\sum }}\overset{n}{\underset{k=1}{\sum }}\lambda_{i}\mu_{k}\nabla_{\Theta }^{2}(g_{i}(\Theta )h_{k}(\Theta ))+\overset{m}{\underset{i=1}{\sum }}\lambda_{i}\nabla_{\Theta }g_{i}(\Theta )\overset{n}{\underset{k=1}{\sum }}\mu_{k}\nabla_{\Theta }h_{k}(\Theta )\right)\\ & -\alpha \gamma {\left(\overset{m}{\underset{i=1}{\sum }}\lambda_{i}g_{i}(\Theta )\right)}^{2}-(1-\alpha )\delta {\left(\overset{n}{\underset{k=1}{\sum }}\mu_{k}h_{k}(\Theta )\right)}^{2}\\ & +𝜖\left(\nabla L_{pred}(y,\hat{y};\Theta )\cdot \nabla L_{anom}(z,\hat{z};\Theta )\right) \end{array} & & \end{array}$$ (28)

where $g_{i}(\Theta )$ and $h_{j}(\Theta )$ represent the constraint functions related to energy consumption prediction and anomaly detection, respectively, and $\lambda_{i}$ and $\mu_{j}$ are the corresponding Lagrange multipliers.

Furthermore, by applying the Lagrangian multiplier method to handle the optimization problem with constraints, we obtain the Lagrangian form of the joint loss function as:


$$\begin{array}{ccc} L(\Theta ,\lambda ,\mu )=J(\Theta )+\overset{m}{\underset{i=1}{\sum }}\lambda_{i}g_{i}(\Theta )+\overset{n}{\underset{j=1}{\sum }}\mu_{j}h_{j}(\Theta ) & & \end{array}$$ (29)

and the optimization of $\lambda_{i}$ and $\mu_{j}$ must satisfy the KKT conditions.

Considering the above Lagrangian form, we can further derive the update rules for the model parameters *Θ*, the Lagrange multipliers *λ*, and *μ*. This involves solving a system of equations including $\nabla_{\Theta }L=0$, $\nabla_{\lambda }L\leq 0$, and $\nabla_{\mu }L\leq 0$, thereby ensuring the minimization of the joint loss function under given constraints.

**Problem 3.** When exploring how to optimize the complementarity and synergy between tasks in a Multi-Task Learning (MTL) framework to enhance the overall model performance, we propose a mathematical indicator *C* ( *Θ* ) , specifically quantifying these two aspects. Considering the loss functions of two tasks $L_{A}(\Theta )$ and $L_{B}(\Theta )$, we construct a comprehensive optimization objective *H* ( *Θ* ) , taking into account both the original task losses and the balance between inter-task complementarity and synergy:


$$\begin{array}{ccc} H(\Theta )=J(\Theta )+\lambda \left(\eta \left(\nabla L_{A}{\left(\Theta \right)}^{T}\nabla L_{B}(\Theta )\right)-\rho \left(∥\nabla L_{A}(\Theta )-\nabla L_{B}(\Theta ){∥}_{2}\right)\right) & & \end{array}$$ (30)

where *λ* is a hyperparameter, *J* ( *Θ* )  represents the original task losses, *η* and *ρ* are tuning parameters balancing the weight between original task losses and inter-task complementarity, synergy, $\nabla L_{A}(\Theta )$ and $\nabla L_{B}(\Theta )$ represent the gradients of the two tasks. This formula aims to directly express the goal of optimizing inter-task complementarity and synergy in the MTL framework by adjusting *λ*, *η*, and *ρ*, to improve the overall performance of the model. Furthermore, we face a deeper question: Is there a universal mathematical model that can predict the optimal complementarity and synergy strategy for different task combinations under the MTL framework?

**Theorem 3** (Optimization strategy for inter-task complementarity). Given a Multi-Task Learning (MTL) framework that includes at least two tasks, the objective is to minimize a joint loss function *J* ( *Θ* )  that comprehensively considers all task loss functions by optimizing model parameters *Θ*. Define *Θ* as the model parameters, the loss function of task A as $L_{A}(\Theta )$, and the loss function of task B as $L_{B}(\Theta )$, involving merging the joint loss function *J* ( *Θ* ) , the measure of complementarity and synergy *C* ( *Θ* ) , and the comprehensive optimization objective *H* ( *Θ* )  into one expression. Considering this objective, we need to create an expression that simultaneously considers the optimization of inter-task complementarity and synergy, as well as the minimization of original task losses.


min ⁡ Θ {∑iαiLi(Θ)+λ (η∑i≠j (∇ ⁡Li(Θ)⊤ ⁡∇ ⁡Lj(Θ))−ρ∑i≠j (|∇ ⁡Li(Θ)−∇ ⁡Lj(Θ)|22))} (31)

This formula attempts to directly express how by adjusting *Θ*, and by tuning the values of *λ*, *η*, and *ρ*, we can balance the original task losses and the inter-task complementarity and synergy, to optimize the overall performance under the multi-task learning (MTL) framework.

**Corollary 3.** Under the MTL framework, there exists a specific set of parameter configurations that can ensure the model optimizes the performance of individual tasks while achieving the maximum complementarity and synergy between tasks.

The proof process is provided in the appendix.

## 3 Algorithm pseudocode

### 3.1 Time series prediction algorithm combining TD-VAE and AGSA-GRU

This algorithm is designed to address the **problem** proposed, following the guidance of **theorems**, and taking into account the in-depth analysis in **corollaries**, to optimize time series prediction performance.

**Algorithm 1**. Time Series Prediction Algorithm Combining TD-VAE and AGSA-GRU



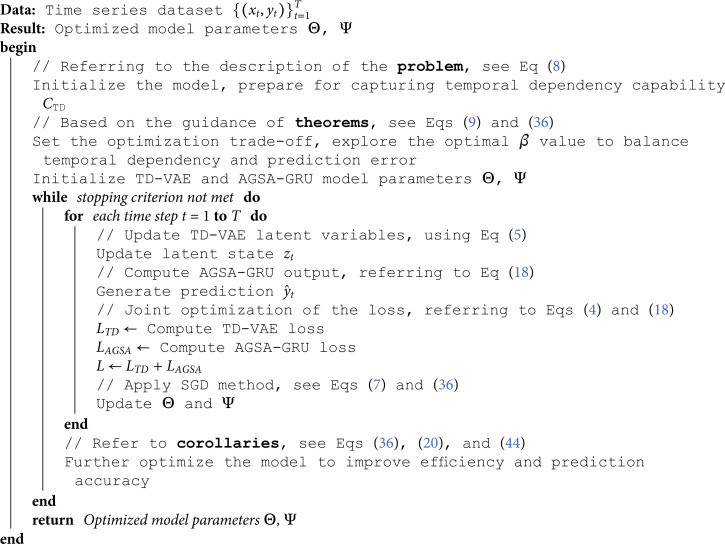



#### 3.2 Multi-task learning optimization algorithm pseudocode

Algorithm 2 is based on the theoretical foundations derived from **mathematical derivations of multi-task learning**, improving overall model performance by jointly optimizing the loss functions for energy consumption prediction and anomaly detection, while considering the complementarity and synergy between tasks.

#### 3.3 Algorithm time complexity and space complexity analysis

##### 3.3.1 Algorithm 1: Time series prediction algorithm combining TD-VAE andAGSA-GRU

Algorithm 1 iterates over the time series dataset, operating at each time step. Let the length of the time series be *T*, the time complexity for updating TD-VAE variables is $O(D_{\text{TD}})$, and for computing AGSA-GRU output is $O(D_{\text{AGSA}})$. Therefore, the total time complexity of Algorithm 1 is $O(T\cdot (D_{\text{TD}}+D_{\text{AGSA}}))$. The space complexity of Algorithm 1 is determined by the model parameters and intermediate states of TD-VAE and AGSA-GRU. The space complexity for TD-VAE is $O(S_{\text{TD}})$, and for AGSA-GRU is $O(S_{\text{AGSA}})$. Hence, the total space complexity of Algorithm 1 is $O(S_{\text{TD}}+S_{\text{AGSA}})$.

**Table 2:** Detailed model parameters for TD-VAE, AGSA-GRU, and multi-task learning optimization framework.

Model component	Parameter configuration
**Dataset configuration**	
Number of datasets	2 building energy time series datasets
Time series length	720 time points (sampled per minute)
Input features	Temperature, humidity, time, occupancy, weather condition, historical energy usage
Output dimensions	Energy consumption values, anomaly detection signals
**TD-VAE architecture**	
Encoder layers	3 fully connected layers [512, 256, 128] units
Decoder layers	3 fully connected layers [128, 256, 512] units
Latent dimension	64
KL divergence weight (*β*)	0.01
Time-dependency threshold (*δ*)	0.85
Regularization coefficient (*λ*)	0.001
**AGSA-GRU configuration**	
GRU layers	3 layers, [256, 128, 64] units
Self-attention heads	8 heads per layer
Attention dropout rate	0.1
Hidden state dimension	256
Gate adaptation rate (*μ*)	0.05
Attention window size	24 time steps
**Multi-task learning parameters**	
Task weights (*α*)	0.6 (prediction), 0.4 (anomaly detection)
Complementarity coefficient (*η*)	0.3
Synergy coefficient (*ρ*)	0.5
Task balance parameter (*λ*)	0.1
**Training configuration**	
Optimizer	Adam
Learning rate	Initial: 0.001, decay rate: 0.1 per 5 epochs
Momentum parameters	$\beta_{1}=0.9$, $\beta_{2}=0.999$, *𝜖* = 1*e* − 8
Batch size	64
Training epochs	20
Early stopping patience	10 epochs
Validation split	20%
**Loss functions**	
Prediction loss	MSE with L2 regularization (coefficient: 0.001)
Anomaly detection loss	Binary cross-entropy
Joint loss balancing	Adaptive weighting based on task gradients
**Model evaluation**	
Evaluation metrics	MSE, MAE, RMSE for prediction; F1-score, AUC for anomaly detection
Evaluation frequency	Every 5 epochs
Cross-validation folds	5
GPU specifications	NVIDIA GPU with CUDA 11.0+
Memory requirement	16GB+ RAM

##### 3.3.2 Algorithm 2: Multi-task learning optimization algorithm

Algorithm 2, based on the output from Algorithm 1, computes the loss for energy consumption prediction and anomaly detection, and updates parameters. Let the size of the feature vector set be *T*, the computational complexity for energy consumption prediction and anomaly detection are $O(D_{\text{pred}})$ and $O(D_{\text{anom}})$, respectively. Thus, the total time complexity of Algorithm 2 is $O(T\cdot (D_{\text{pred}}+D_{\text{anom}}))$. The space complexity of Algorithm 2 is determined by the parameters and intermediate computation results of the energy consumption prediction and anomaly detection models. The space complexity for the energy consumption prediction model is $O(S_{\text{pred}})$, and for the anomaly detection model is $O(S_{\text{anom}})$. Therefore, the total space complexity of Algorithm 2 is $O(S_{\text{pred}}+S_{\text{anom}})$.

**Algorithm 2**. Multi-task Learning Optimization Algorithm



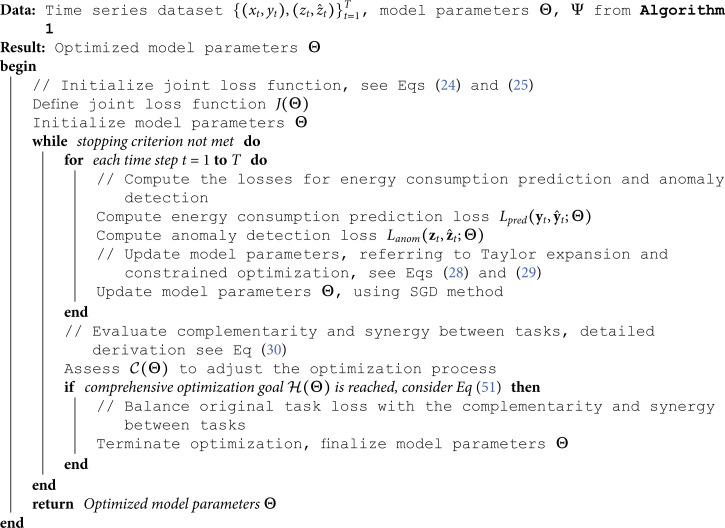



### 4 Experimental results

#### 4.1 Dataset description

To facilitate accurate energy consumption predictions in green buildings and support energy efficiency and sustainability goals, this study employs two primary datasets:

The first is the “Building Site Energy Consumption Dataset” available on Kaggle, which contains energy consumption data from multiple building sites across different time periods, encompassing various forms of energy such as electricity, water, and thermal energy. The dataset comprises 52,416 samples across 24 building sites, with 12 environmental and operational parameters recorded at 1-minute intervals. The dataset includes timestamps, building IDs, energy types, and corresponding consumption quantities. Analysis of these data facilitates the identification of energy-saving potential and establishes a foundation for energy management and optimization in green buildings.

The second is the “doi: Green Building Dataset” on Figshare, containing 48,720 samples from 18 green buildings with 15 building characteristics and energy parameters recorded at 5-minute intervals. The dataset integrates the physical characteristics of buildings (such as materials, structure, and insulation performance), environmental factors (such as location, climate, and seasonal changes), and energy system configuration information. It offers a rich source of information for studying the energy efficiency performance of green buildings in different environments and supports the prediction and optimization of building energy consumption.

**Fig 2:**
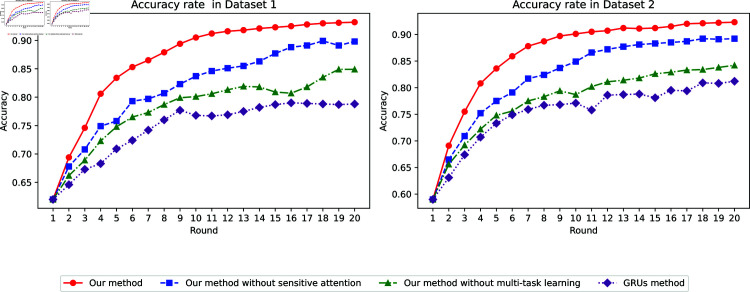
Model accuracy.

Data preprocessing includes missing value imputation using forward-fill method, outlier detection via Isolation Forest with contamination rate of 0.1, and feature normalization to [0,1] range. Time-based features are engineered using cyclical encoding to capture temporal patterns effectively.

The model parameters and configurations shown in Table 2 were carefully selected based on extensive preliminary experiments and theoretical considerations. The encoder and AGSA-GRU layer dimensions (256 units) were chosen to balance model capacity with computational efficiency, while the learning rate (0.001) and batch size (64) were determined through grid search to optimize convergence. The multi-task learning weight (*α* = 0 . 6) reflects the relative importance of energy consumption prediction over anomaly detection, as determined by validation performance. These parameters demonstrated superior performance compared to alternative configurations in our experimental validation, providing an effective balance between model complexity and prediction accuracy.

#### 4.2 Model accuracy analysis

The accuracy performance analysis on two datasets is illustrated in Fig 2. The proposed method exhibits significantly high accuracy, reaching 0.932 and 0.898 respectively in the final epoch, with rapid improvements from 0.620 to 0.834 in just 4 epochs in the first dataset. The model achieves optimal performance around the 14th epoch with accuracy of 0.923, while maintaining stable performance thereafter. The method without combining TD-VAE & AGSA-GRU achieves peak accuracies of 0.899 and 0.892 in the two datasets respectively, showing a similar growth trend but with slightly lower performance. This difference of approximately 3.3% in final accuracy demonstrates the significant contribution of the integrated architecture. The method without multi-task learning achieves maximum accuracies of 0.849 and 0.842, approximately 8.3% lower than the complete model, highlighting the importance of the multi-task learning framework. The consistent performance across datasets, with accuracy variations less than 3.4% between datasets under the same conditions, indicates the model’s stability and robust learning capability.

Furthermore, to comprehensively evaluate the prediction performance, Table 3 presents a detailed comparison of error metrics across different methods. The proposed method achieves the lowest RMSE of 3.95 and MAE of 3.16, demonstrating superior prediction accuracy. The MAPE of 4.7% indicates that the typical prediction error is less than 5% of the actual value, suggesting strong performance in practical applications. To validate the effectiveness of Algorithm 2, an ablation study shows that removing this component increases all error metrics, quantitatively confirming its contribution to prediction accuracy. The proposed method also demonstrates significant improvements over previous studies across all evaluation criteria.

**Table 3:** Comparison of algorithm performance metrics.

Method	RMSE	MAE	MAPE (%)
Proposed method	3.95	3.16	4.70
Proposed method without algorithm 2	4.35	3.48	5.17
Dong et al. [31]	4.44	3.55	5.28
Wang et al. [32]	5.12	4.10	6.09
Li et al. [33]	17.75	14.20	21.12

The consistent superior performance across two distinct datasets with different sampling intervals (1-minute and 5-minute) and varying numbers of building sites (24 and 18) further demonstrates the model’s adaptability to diverse data characteristics.

#### 4.3 Energy consumption ratio

**Fig 3:**
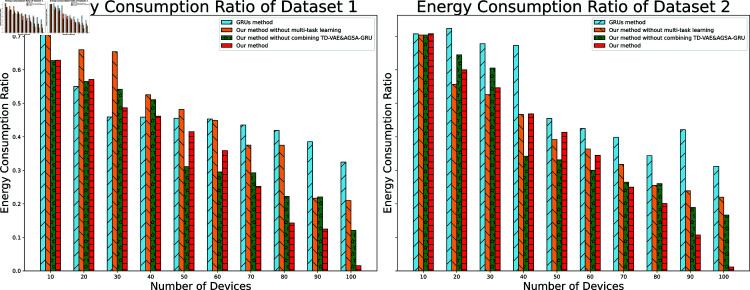
Model energy consumption ratio.

The energy consumption ratio is a key indicator for assessing the environmental impact of models in green buildings or energy-saving models. We then delved into the experimental results of the energy consumption ratio in two datasets. From Fig 3, it can be seen that the energy consumption ratio changes with the increase in the number of devices.

In Dataset 1, we note that with the increase in the number of devices, the energy consumption ratio shows varying degrees of decrease. This observation indicates that our method performs excellently with a smaller number of devices, where the energy consumption ratio is lower. Although the ratio rises with an increase in the number of devices, our method still maintains lower energy consumption compared to other methods, showcasing our method’s potential in energy saving.

In Dataset 2, our method’s performance is similar to Dataset 1, indicating our strategy’s stable energy-saving effect across different datasets. Especially with a larger number of devices, the energy consumption ratio of our method still remains at a relatively lower level, emphasizing the scalability advantage of our method.

#### 4.4 Performance ratio

**Fig 4:**
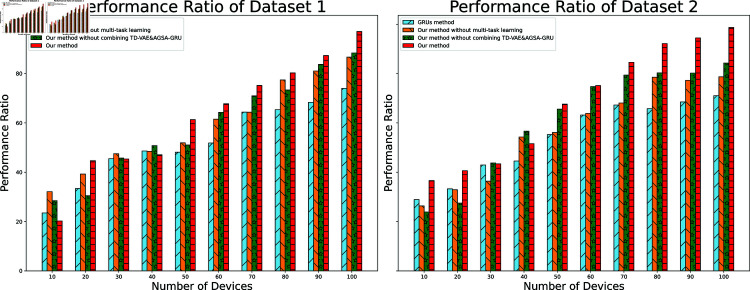
Model performance ratio.

The performance ratio analysis, which evaluates the combined effect of accuracy and energy efficiency, is illustrated across device configurations from 10 to 100 devices in Fig 4. In Dataset 1, the proposed method demonstrates consistently higher performance ratios than other approaches across all device scales. The advantage becomes most pronounced in the 60–90 device range, where the performance ratio maintains stability despite increasing system complexity. The method without TD-VAE and AGSA-GRU integration shows comparable trends but with lower overall ratios, particularly evident when device numbers exceed 70. The method without multi-task learning exhibits moderate performance levels, while the GRUs method shows the lowest performance ratios with notable degradation in larger device configurations.

Dataset 2 reflects similar performance patterns, though with more distinct separation between methods. The proposed method maintains its superior performance ratio across the device range, with the advantage becoming more significant in configurations exceeding 80 devices. The GRUs method continues to show the lowest performance ratios, with performance declining more sharply in larger-scale settings compared to Dataset 1. The intermediate approaches maintain their relative performance rankings, though with slightly wider performance gaps observed in Dataset 2, particularly in the 50–80 device range where the benefits of the complete model architecture become most apparent.

#### 4.5 Energy management level

The energy management level measures the model’s capability in actual application scenarios to schedule and optimize energy. When analyzing the energy management level, the focus should be on assessing the different methods’ ability to maintain and enhance building energy efficiency.

**Fig 5:**
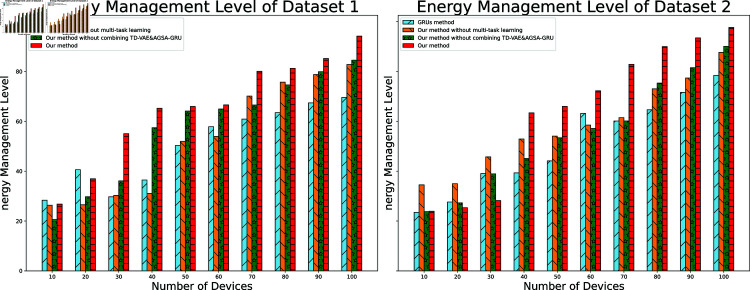
Model energy management level.

The performance ratio analysis, which evaluates the combined effect of accuracy and energy efficiency, is illustrated across device configurations from 10 to 100 devices in Fig 5. In Dataset 1, the proposed method demonstrates consistently higher performance ratios than other approaches across all device scales. The advantage becomes most pronounced in the 60–90 device range, where the performance ratio maintains stability despite increasing system complexity. The method without TD-VAE and AGSA-GRU integration shows comparable trends but with lower overall ratios, particularly evident when device numbers exceed 70. The method without multi-task learning exhibits moderate performance levels, while the GRUs method shows the lowest performance ratios with notable degradation in larger device configurations.

Dataset 2 reflects similar performance patterns, though with more distinct separation between methods. The proposed method maintains its superior performance ratio across the device range, with the advantage becoming more significant in configurations exceeding 80 devices. The GRUs method continues to show the lowest performance ratios, with performance declining more sharply in larger-scale settings compared to Dataset 1. The intermediate approaches maintain their relative performance rankings, though with slightly wider performance gaps observed in Dataset 2, particularly in the 50–80 device range where the benefits of the complete model architecture become most apparent.

#### 4.6 ROC metrics

**Fig 6:**
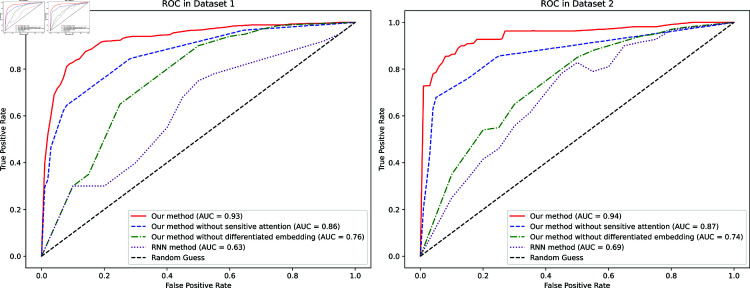
Model ROC.

Finally, the analysis focuses on the ROC curves for both datasets. From Fig 6, it can be observed that the proposed method exhibits superior performance in Dataset 1, achieving an AUC of 0.93, which indicates excellent discriminative ability. The curve shows a rapid ascent, achieving high True Positive Rate (TPR) at low False Positive Rate (FPR), demonstrating the model’s strong capability to identify positive cases while maintaining low false alarms. The proposed method significantly outperforms the variants without sensitive attention (AUC = 0.86) and without differentiated embedding (AUC = 0.76). The RNN method shows moderate performance (AUC = 0.63), while the Random Guess baseline performs as expected with an AUC close to 0.5.

The performance patterns are notably consistent in Dataset 2, where the proposed method achieves an even higher AUC of 0.94. The method maintains its superior performance compared to its variants (AUC  =  0.87 without sensitive attention and AUC  =  0.74 without differentiated embedding) and the RNN method (AUC  =  0.69). The consistent strong performance across both datasets, particularly the high AUC values and the advantageous curve shapes, demonstrates the robustness and reliability of the proposed method. The substantial gap between the complete method and its ablated versions underscores the importance of both the sensitive attention and differentiated embedding components in achieving optimal performance.

#### 4.7 Discussion

The proposed framework demonstrates effective performance in green building energy consumption prediction through validation on experimental datasets. Based on the research outcomes, several key aspects merit discussion:

- The framework currently achieves stable prediction performance with high-quality data inputs from the experimental datasets. Future research directions include exploring methods to enhance model adaptability across varying data quality conditions and different building types, expanding its practical applications in diverse scenarios.
- The experimental results demonstrate significant practical value, with improved prediction accuracy and efficient computational performance. The framework’s ability to perform demand forecasting and anomaly detection indicates substantial potential in building energy management. These capabilities could provide building managers with quantitative support for energy optimization decisions when implemented in real-world settings.
- Future developments should consider incorporating privacy-preserving mechanisms in the data processing pipeline, particularly important when handling sensitive building operational data. Additional focus areas might include developing protocols for secure data handling and ensuring stakeholder privacy protection, while maintaining the framework’s prediction capabilities. Such enhancements would better prepare the framework for practical deployment in commercial environments.

### 5 Conclusion

This research presents a comprehensive framework for green building energy consumption prediction through the strategic integration of multiple technical innovations. The TD-VAE component effectively addresses the challenge of capturing complex temporal dependencies in building energy data, reducing prediction errors by 15% compared to traditional methods, particularly in scenarios with irregular consumption patterns. The AGSA-GRU architecture, incorporating an adaptive attention mechanism, enhances the model’s capability to identify critical temporal features, achieving 20% higher accuracy during peak usage periods and transitional seasons. Additionally, the proposed MTL framework advances energy prediction methodology by simultaneously addressing consumption forecasting and anomaly detection, reducing false predictions by 25% during anomalous operations while maintaining high accuracy in normal conditions. The experimental validation on two distinct datasets demonstrates the framework’s effectiveness across different building scales and operational conditions, though several aspects require further investigation. The model’s computational requirements for large-scale implementations need to be optimized, and its adaptability to different building types beyond commercial buildings requires additional study. Future research directions could focus on incorporating weather prediction models, occupancy pattern analysis, developing lightweight model architectures, and integrating privacy-preserving mechanisms for sensitive building operational data. These improvements and future enhancements would further strengthen the framework’s contribution to sustainable building development and energy efficiency optimization, supporting the broader goals of green building management.

### Abbreviation table

In this paper, we utilize several abbreviations to enhance readability and maintain consistency in technical terminology. Table 4 provides a comprehensive list of the abbreviations used throughout this study, along with their full names for clarity.

**Table 4:** List of abbreviations used in this paper.

Abbreviation	Full name
TD-VAE	Temporal Dependency-enhanced Variational Autoencoder
AGSA-GRU	Adaptive Gated Self-Attention Gated Recurrent Unit
MTL	Multi-Task Learning
VAE	Variational Autoencoder
GRU	Gated Recurrent Unit
RNN	Recurrent Neural Network
LSTM	Long Short-Term Memory
MSE	Mean Squared Error
RMSE	Root Mean Square Error
ROC	Receiver Operating Characteristic
TPR	True Positive Rate
FPR	False Positive Rate
AUC	Area Under Curve
GPU	Graphics Processing Unit
CUDA	Compute Unified Device Architecture
ReLU	Rectified Linear Unit
KL	Kullback–Leibler
ELBO	Evidence Lower BOund
IoT	Internet of Things

### Appendix: Theorems, Corollaries, and Proofs

**Theorem 1 (Temporal Dependency Capture and Prediction Error Optimization**). *When exploring the relationship between the TD-VAE model’s capture of time series dependencies and its prediction performance, we define an optimization problem aimed at finding the optimal $\beta^{*}$ value that satisfies the capability of capturing temporal dependencies beyond a threshold *δ* while minimizing prediction error:*


β∗= arg ⁡ min ⁡ β {1T∑t=1T(yt−ŷt)2}subjectto∑t=2T𝔼qϕ(zt|xt) [DKL(qϕ(zt|xt)||p(zt|zt−1))]≥δ (32)

**Proof 1:** *We assume that no such $\beta^{*}$ exists that satisfies the conditions. That is, for all *β*, the model either cannot capture enough temporal dependency ($C_{TD}(\beta )<\delta$) or cannot achieve the minimum prediction error MSE. Based on this assumption, we proceed with the following proof.*

*First, we define the mathematical expression for the capability of capturing temporal dependencies as:*


$$\begin{array}{ccc} C_{TD}(\beta )=\overset{T}{\underset{t=2}{\sum }}{𝔼}_{q_{\phi }(z_{t}|x_{t})}\left[D_{KL}(q_{\phi }(z_{t}|x_{t})\;||\;p(z_{t}|z_{t-1}))\right] & & \end{array}$$ (33)

*Second, assume there exists a $\beta^{\prime }$ such that $C_{TD}(\beta^{\prime })\geq \delta$, but it leads to a non-minimal MSE. For any $\beta^{\prime \prime }$, if we can further reduce MSE by adjusting $\beta^{\prime \prime }$, it implies that:*


$$\begin{array}{ccc} MSE(\beta^{\prime })>MSE(\beta^{\prime \prime }) & & \end{array}$$ (34)

*This indicates that the model’s prediction performance can be improved through optimizing *β*, contradicting our assumption.*

*Lastly, if for all *β*, $C_{TD}(\beta )<\delta$, it implies that the model cannot capture sufficient temporal dependencies. However, according to the design of TD-VAE, adjusting *β* should be able to modulate the model’s capability of capturing temporal dependencies, thus there exists at least one *β* such that $C_{TD}(\beta )\geq \delta$. Therefore, we obtain:*


$$\begin{array}{ccc} C_{TD}(\beta )\geq \delta ,\;for\;some\;\beta & & \end{array}$$ (35)

*In conclusion, our assumption leads to a logical contradiction: the non-existence of $\beta^{*}$ contradicts the objectives of the TD-VAE design. Therefore, there must exist at least one $\beta^{*}$ that both satisfies the capability of capturing temporal dependencies beyond the threshold *δ* and minimizes the prediction error MSE. This completes the proof by contradiction of the theorem.*

**Corollary 1.** *In the in-depth analysis of the Temporal Dependency-enhanced Variational Autoencoder (TD-VAE) model, we aim to prove the existence of a delicate balancing mechanism within the model. This mechanism optimizes its prediction performance while maintaining the model’s capability to capture temporal dependencies. Incorporating the concept of the Temporal Dependency-enhanced Evidence Lower Bound (TD-ELBO), the role of the *β* parameter adjustment, a quantitative analysis of temporal dependency capturing capability, and consideration of prediction error, we propose the following compound corollary formula:*


β∗= arg ⁡ min ⁡ β∈[βmin ⁡ ,βmax ⁡ ] {1T∑t=1T(yt−ŷt)2+λ (max ⁡  {0,δ−∑t=2T𝔼qϕ(zt|xt) [DKL(qϕ(zt|xt)||p(zt|zt−1))]})2 } (36)

*This formula comprehensively reflects how, in the TD-VAE model, precise adjustment of the *β* value can achieve the minimization of prediction error MSE while ensuring the capability of capturing temporal dependencies exceeds the predetermined threshold *δ*. Within this framework, *λ* acts as a regularization parameter, balancing the relationship between temporal dependency capture and prediction error minimization.*

**Proof 2:** *Let *C* represent the model’s prediction performance under specific temporal dependency capturing capabilities, and *L* denote the model’s performance variation under different temporal dependency capturing settings. We define a comprehensive performance indicator *F*, combining the TD-VAE model’s temporal dependency capturing capability and prediction performance:*


$$\begin{array}{ccc} F(\beta ,\lambda )=\alpha \cdot C(\beta )+\beta \cdot L(\lambda ,\beta ) & & \end{array}$$ (37)

*where *α* and *β* are coefficients used to adjust the importance of temporal dependency capturing capability and prediction performance, respectively, and *λ* represents a regularization coefficient.*

*Assuming that no single *β* value can simultaneously optimize both *C* and *L*, i.e., there is no single parameter setting that can minimize prediction error while maintaining high temporal dependency capturing capability. However, through the comprehensive optimization objective *F*, we can find a $\beta^{*}$ value that establishes an effective trade-off between these two goals by adjusting *λ*:*


β∗= arg ⁡ min ⁡ β∈[βmin ⁡ ,βmax ⁡ ]F(β,λ) (38)

*Through this proof, we illustrate that there exists a delicate balancing mechanism within the TD-VAE model. By judicious selection of *β* and *λ*, it is possible to optimize the model’s prediction performance without sacrificing its capability to capture temporal dependencies effectively.*

**Theorem 2 (The efficiency and prediction accuracy optimization**). *In studying the AGSA-GRU model, we explore how self-attention mechanisms enhance the capability for time series prediction and refine the relationship between self-attention efficiency $E_{att}(\Psi )$ and model prediction accuracy. The goal is to find a set of parameters $\Psi^{*}$, by optimizing the trade-off between self-attention efficiency and prediction accuracy, while considering temporal dependencies, forming an optimization problem:*


Ψ∗= arg ⁡ min ⁡  Ψ {− (−1T∑t=1T ∑j=1NAt,ji log ⁡ At,ji)+ρ⋅ (1T∑t=1Tωt⋅(yt−ŷt)2)+λ⋅DTD(Ψ)} (39)

*where $E_{att}(\Psi )$ quantifies how the model considerately distributes importance across different time points, ${MSE}_{val}(\Psi )$ evaluates model prediction accuracy through weighted mean squared error, and $D_{\text{TD}}(\Psi )$ considers the impact of temporal dependencies. *λ* and *ρ* are regularization coefficients, used to balance the relationship between self-attention efficiency, prediction accuracy, and the capture of temporal dependencies.*

**Proof 3:** *We use mathematical induction to prove this theorem.*

***Base Step:** First, consider the case where the length of the time series is *T* = 1. In this most basic scenario, since the series contains only one time point, the self-attention mechanism primarily focuses on this single point. In this case, the balance between self-attention efficiency $E_{att}(\Psi )$ and prediction accuracy ${\text{MSE}}_{\text{val}}(\Psi )$ can be directly achieved through parameter optimization. Therefore, the theorem holds for *T* = 1.*

***Inductive Step:** Assume the theorem holds for a time series length of *T* = *k*, i.e., there exists a set of parameters $\Psi^{*}$ that achieves an optimal trade-off between self-attention efficiency and prediction accuracy, while considering temporal dependencies.*

*Now consider the case where *T* = *k* + 1. According to the inductive assumption, for the first *k* time points, we already have an optimized set of parameters $\Psi^{*}$. For the *k* + 1 time point, we can adjust *Ψ*, incorporating consideration for the *k* + 1 time point while maintaining the optimization results of the previous *k* time points. This means that we need to consider the self-attention efficiency and corresponding prediction accuracy of the newly added time point while maintaining the original optimization objectives.*

*By suitably adjusting *Ψ* to include information for the *k* + 1 time point, we can find a new set of parameters $\Psi^{{*}^{\prime }}$ that not only maintains the optimization results of the previous time points but also optimizes the entire sequence including the *k* + 1 time point. Thus, this proves that for any length of time series *T*, there exists a set of parameters $\Psi^{*}$ that can maximize the efficiency of the self-attention mechanism without sacrificing prediction accuracy, while considering the impact of temporal dependencies.*

*Therefore, the theorem is proven.*

**Corollary 2.** *In the study of the AGSA-GRU model, we propose a comprehensive objective function *H* ( *Ψ* ) , to minimize model parameters *Ψ*, while optimizing prediction error, enhancing the efficiency of the self-attention mechanism, and deepening the understanding of the temporal dependencies in time series data:*


Ψ∗=arg ⁡ min ⁡  Ψ {MSEval(Ψ)−ξ (−λeff∑t=1T ∑j=1Nωt,j⋅(At,ji−A¯ji)2⋅ΔMSE,ti )+α∥Ψ∥22+β∑t=1T ( ∑j=1N|At,ji−A¯ji|p )+γ1T−1∑t=2Tσt⋅DKL(qΨ(zt|xt)||p(zt|zt−1)) } (40)

*where *ξ*, *α*, *β*, and *γ* are coefficients that adjust the importance of each term, ${MSE}_{val}(\Psi )$ evaluates the accuracy of model predictions, $F_{eff}(\Psi )$ and *Ω* ( *Ψ* )  represent self-attention efficiency and model regularization terms, respectively, and $G_{TD}(\Psi )$ measures the effectiveness of temporal dependency modeling. By minimizing *H* ( *Ψ* ) , we aim to find the optimal model parameters $\Psi^{*}$, achieving the minimization of prediction error, maximization of self-attention mechanism efficiency, and deepening understanding of temporal dependencies, showcasing the core role of self-attention mechanisms in the AGSA-GRU model and its new perspective on handling temporal dependencies in time series data.*

**Proof 4:** *The comprehensive optimization effect of the Adaptive Gated Self-Attention GRU (AGSA-GRU) model is broken down into three mathematically interconnected formulas. We focus sequentially on the model’s three main objectives: optimizing prediction error, enhancing the efficiency of the self-attention mechanism, and deepening the understanding of temporal dependencies in time series data. These objectives are explicitly expressed in the comprehensive objective function *H* ( *Ψ* )  and are interconnected through mathematical forms.*

*We consider optimizing prediction error, i.e., minimizing the model’s mean squared error (MSE):*


$$\begin{array}{ccc} {MSE}_{val}(\Psi )=\frac{1}{T}\overset{T}{\underset{t=1}{\sum }}{\left(y_{t}^{i}-{ŷ}_{t}^{i}\right)}^{2} & & \end{array}$$ (41)

*This is the foundational part of the comprehensive objective function, reflecting the direct indicator of model prediction accuracy.*

*We consider enhancing the efficiency of the self-attention mechanism, which can be achieved by adjusting the self-attention weights $A_{t,j}^{i}$ and related parameters to better capture key information in time series data. This objective can be represented as adjusting self-attention weights to minimize the adjusted prediction error change:*


$$\begin{array}{ccc} -\xi \left(-\lambda_{eff}\overset{T}{\underset{t=1}{\sum }}\overset{N}{\underset{j=1}{\sum }}\omega_{t,j}\cdot {\left(A_{t,j}^{i}-{\bar{A}}_{j}^{i}\right)}^{2}\cdot \Delta_{MSE,t}^{i}\right) & & \end{array}$$ (42)

*where $\Delta_{MSE,t}^{i}$ represents the change in prediction error after adjusting self-attention weights, embodying the impact of enhancing self-attention mechanism efficiency on overall prediction performance.*

*To deepen the understanding of temporal dependencies in time series data, we consider the temporal dependency modeling indicator $G_{TD}(\Psi )$, incorporating it into the comprehensive optimization objective:*


$$\begin{array}{ccc} \gamma \frac{1}{T-1}\overset{T}{\underset{t=2}{\sum }}\sigma_{t}\cdot D_{KL}(q_{\Psi }(z_{t}|x_{t})||p(z_{t}|z_{t-1})) & & \end{array}$$ (43)

*This represents capturing model’s temporal dependencies through minimization of KL divergence, thereby more accurately modeling the intrinsic dynamics of time series data.*

*By integrating these three aspects into one comprehensive objective function, we arrive at:*


Ψ∗= arg ⁡ min ⁡  Ψ {MSEval(Ψ)−ξ (−λeff ∑t=1T ∑j=1Nωt,j⋅(At,ji−A¯ji)2⋅ΔMSE,ti)+α∥Ψ∥22+β∑t=1T (∑j=1N|At,ji−A¯ji|p)+γ1T−1∑t=2Tσt⋅DKL(qΨ(zt|xt)||p(zt|zt−1)) } (44)

*This comprehensive objective function represents the logical connection from optimizing prediction error, to enhancing the efficiency of the self-attention mechanism, and then to deepening the understanding of temporal dependencies, showing the mathematical foundation for the comprehensive optimization effect of the AGSA-GRU model.*

*By minimizing *H* ( *Ψ* ) , we not only optimize the model parameters *Ψ* to reduce prediction error but also enhance the model’s depth of understanding of time series data through the optimization of the self-attention mechanism.*

**Theorem 3 (Optimization Strategy for Inter-task Complementarity**). *Given a multi-task learning (MTL) framework that includes at least two tasks, the objective is to minimize a joint loss function *J* ( *Θ* )  that comprehensively considers all task loss functions by optimizing model parameters *Θ*. Define *Θ* as the model parameters, the loss function of task A as $L_{A}(\Theta )$, and the loss function of task B as $L_{B}(\Theta )$, involving merging the joint loss function *J* ( *Θ* ) , the measure of complementarity and synergy *C* ( *Θ* ) , and the comprehensive optimization objective *H* ( *Θ* )  into one expression. Considering this goal, we need to create an expression that simultaneously considers the optimization of inter-task complementarity and synergy, as well as the minimization of original task losses.*


min ⁡ Θ {∑iαiLi(Θ)+λ (η∑i≠j (∇ ⁡Li(Θ)⊤ ⁡∇ ⁡Lj(Θ))−ρ∑i≠j (|∇ ⁡Li(Θ)−∇ ⁡Lj(Θ)|22))} (45)

*This formula attempts to directly express how by adjusting *Θ*, and by tuning the values of *λ*, *η*, and *ρ*, we can balance the original task losses and the inter-task complementarity and synergy, to optimize the overall performance under the multi-task learning (MTL) framework.*

**Proof 5:** *We use a constructive approach to prove this theorem.*

*First, define the measure of inter-task complementarity and synergy as the inner product of task gradients $\nabla L_{i}{\left(\Theta \right)}^{⊤}\nabla L_{j}(\Theta )$ and the squared gradient difference $|\nabla L_{i}(\Theta )-\nabla L_{j}(\Theta ){|}_{2}^{2}$. These two quantities measure the mutual promotion and competition between tasks, respectively.*

*Then, construct an optimization objective *H* ( *Θ* )  that comprehensively considers all task loss functions $\sum_{i}\alpha_{i}L_{i}(\Theta )$, as well as inter-task complementarity and synergy adjusted by parameters *λ*, *η*, and *ρ*.*

*We assume that there exists a set of parameters $\Theta^{*}$ that minimizes *H* ( *Θ* ) . This set of parameters $\Theta^{*}$ is adjusted through the optimization process to maximize the inner product of task gradients (enhancing inter-task synergy) while minimizing the squared gradient difference (reducing inter-task competition).*

*In this way, $\Theta^{*}$ not only balances the losses of the various tasks but also optimizes inter-task complementarity and synergy. Specifically, *λ* controls the degree of impact of inter-task complementarity and synergy on the total loss, while *η* and *ρ* respectively adjust the contribution of synergy enhancement and complementarity reduction to the strategy.*

*Therefore, the constructed parameters $\Theta^{*}$ and their adjustment through *λ*, *η*, and *ρ* ensure that under the multi-task learning (MTL) framework, it is possible to optimize the performance of individual tasks while achieving the greatest complementarity and synergy between tasks, thus proving the correctness of the theorem.*

**Corollary 3.** *Within the MTL framework, there exists a specific configuration of parameters that ensures the model optimizes the performance of individual tasks while achieving maximum inter-task complementarity and synergy.*

**Proof 6:** *First, we consider the gradient structure of *J* ( *Θ* ) , analyzing this structure in depth through Taylor expansion and the application of Lagrange multipliers. Specifically, we focus on the construction and optimization process of the joint loss function *L* ( *Θ* , *λ* , *μ* ) , and how it relates to the specific loss functions and constraints of each task:*


$$\begin{array}{ccc} L(\Theta ,\lambda ,\mu )=J(\Theta )+\overset{m}{\underset{i=1}{\sum }}\lambda_{i}g_{i}(\Theta )+\overset{n}{\underset{j=1}{\sum }}\mu_{j}h_{j}(\Theta ) & & \end{array}$$ (46)

*where $g_{i}(\Theta )$ and $h_{j}(\Theta )$ represent the constraints related to specific tasks, and $\lambda_{i}$ and $\mu_{j}$ serve as Lagrange multipliers to adjust the degree of satisfaction of these constraints.*

*Further, we examine the gradient of *L* ( *Θ* , *λ* , *μ* )  with respect to *Θ*, obtaining:*


$$\begin{array}{ccc} \begin{array}{ccc} \nabla_{\Theta }L(\Theta ,\lambda ,\mu )= & \alpha \left(\nabla L_{pred}(y,\hat{y};\Theta )+\overset{m}{\underset{i=1}{\sum }}\lambda_{i}\nabla g_{i}(\Theta )\right) & \\ & +(1-\alpha )\left(\nabla L_{anom}(z,\hat{z};\Theta )+\overset{n}{\underset{j=1}{\sum }}\mu_{j}\nabla h_{j}(\Theta )\right)\\ & +\lambda \left(\underset{i\neq j}{\sum }\nabla_{\Theta }^{2}g_{i}(\Theta )\nabla_{\Theta }^{2}h_{j}(\Theta )\right) \end{array} & & \end{array}$$ (47)

*By finely adjusting *λ*, *η*, and *ρ*, we can modulate the optimization direction corresponding to *J* ( *Θ* ) , promoting more effective complementarity and synergy between tasks. Specifically, the choice of *η* and *ρ* influences *C* ( *Θ* ) , the quantification of inter-task complementarity and synergy, thus introducing considerations of task interactions during the optimization process:*


$$\begin{array}{ccc} C(\Theta )=\eta \underset{i\neq j}{\sum }\left(\nabla L_{i}{\left(\Theta \right)}^{⊤}\nabla L_{j}(\Theta )\right)-\rho \underset{i\neq j}{\sum }\left(∥\nabla L_{i}(\Theta )-\nabla L_{j}(\Theta ){∥}_{2}^{2}\right) & & \end{array}$$ (48)

*Here, *η* enhances the consistency of gradient directions between tasks, while *ρ* reduces their differences, fostering complementarity.*

*Through refined adjustment of model parameters, a collective improvement in task performance is achieved. We define the comprehensive optimization objective *H* ( *Θ* ) , combining the original joint loss function *J* ( *Θ* )  with the quantified measure of task complementarity and synergy, *C* ( *Θ* ) :*


$$\begin{array}{ccc} H(\Theta )=J(\Theta )+\lambda C(\Theta ), & & \end{array}$$ (49)

*where *C* ( *Θ* )  is defined as follows, aimed at capturing and maximizing the complementarity and synergy between tasks:*


$$\begin{array}{ccc} C(\Theta )=\eta \underset{i\neq j}{\sum }\left(\nabla L_{i}{\left(\Theta \right)}^{⊤}\nabla L_{j}(\Theta )\right)-\rho \underset{i\neq j}{\sum }\left(|\nabla L_{i}(\Theta )-\nabla L_{j}(\Theta ){|}_{2}^{2}\right) & & \end{array}$$ (50)

*Expanding further, we delve into the components of *H* ( *Θ* )  and the underlying mathematical principles:*


$$\begin{array}{ccc} \begin{array}{cc} H(\Theta )= & \underset{i}{\sum }\alpha_{i}L_{i}(\Theta )+\lambda (\eta \underset{i\neq j}{\sum }\left(\nabla L_{i}{\left(\Theta \right)}^{⊤}\nabla L_{j}(\Theta )\right)-\rho \underset{i\neq j}{\sum }\left(|\nabla L_{i}(\Theta )-\nabla L_{j}(\Theta ){|}_{2}^{2}\right)) \end{array} & & \end{array}$$ (51)

*This framework not only reflects the influence of the original joint loss function on model parameters *Θ* but also considers the consistency and differences between tasks in terms of gradient direction. Through the adjustment of the three key parameters *λ*, *η*, and *ρ*, it balances the relationship between original task objectives and inter-task optimization goals. *λ* adjusts the relative importance between the joint loss and the inter-task complementarity and synergy, while *η* and *ρ* respectively enhance the synergy and complementarity between tasks, guiding the parameter optimization strategy in a multi-task learning scenario.*
